# Development of a bead-based multiplex assay to quantify bovine interleukin-10, tumor necrosis factor-α, and interferon-γ concentrations in plasma and cell culture supernatant

**DOI:** 10.3168/jdsc.2021-0191

**Published:** 2022-03-03

**Authors:** Anja Sipka, Sabine Mann, Susanna Babasyan, Heather Freer, Bettina Wagner

**Affiliations:** Department of Population Medicine and Diagnostic Sciences, College of Veterinary Medicine, Cornell University, Ithaca, NY 14853

## Abstract

The quantification of cytokines can improve our understanding of immune response and inflammation dynamics in dairy cows. Bead-based assays provide a sensitive, high-throughput platform, allowing for simultaneous quantification of multiple cytokines within a wide linear detection range. Our objective was to develop a multiplex bead-based assay using monoclonal antibodies for simultaneous quantification of bovine tumor necrosis factor (TNF)-α, IL-10, and IFN-γ in plasma and peripheral blood mononuclear cell (PBMC) culture supernatants. Recombinant cytokine standards produced in mammalian cells were used to determine the lower limit of detection and the linear detection range for each cytokine. The lower limit of detection was 110 pg/mL for IL-10, 95 pg/mL for TNF-α, and 20 pg/mL for IFN-γ. The linear quantification range was 110 to 241,000 pg/mL for IL-10, 95 to 620,000 pg/mL for TNF-α, and 20 to 130,000 pg/mL for IFN-γ. All 3 monoclonal capture and detection antibodies were specific for their respective cytokine analyte when using the recombinant IL-10, TNF-α, and IFN-γ standards. Intraassay and interassay coefficients of variation (CV) were <10% and <12%, respectively, for all analytes and samples matrices. Next, concentrations of native cytokines were determined in PBMC culture supernatants (n = 4) and in plasma from whole-blood samples (n = 6) with or without stimulation with *Escherichia coli* lipopolysaccharide or a mix of phorbol myristate acetate (PMA) and ionomycin. Peak concentrations of all 3 cytokines were secreted from PBMC after PMA/ionomycin stimulation (TNF-α, 8 h, range: 39,266–506,422 pg/mL; IL-10, 18 h, range: 15,770–63,415 pg/mL; IFN-γ 18 h, range: 189,977–492,659 pg/mL). In contrast, the highest concentrations in plasma from whole-blood stimulation were observed for IL-10 and TNF-α after LPS stimulation (TNF-α, 4 h, range: 1,764–13,460 pg/mL; IL-10, 24 h, range: 2,401–6,371 pg/mL), whereas PMA and ionomycin induced the highest secretion of IFN-γ (18 h, range: 53–20,215 pg/mL). In conclusion, the multiplex assay can quantify native IL-10, TNF-α, and IFN-γ across a broad concentration range in bovine plasma and cell culture supernatant, thereby providing a novel tool to evaluate inflammatory profiles in cattle and especially in dairy cows with inflammatory conditions. The existing multiplex assay can be expanded in the future by adding bead assays for additional bovine cytokines.

## Highlights

• Bead-based assays provide a platform for simultaneous quantification of multiple cytokines.
• We developed a multiplex bead-based assay for quantification of bovine TNF-α, IL-10, and IFN-γ to evaluate inflammatory profiles in cattle.
• We used mononuclear reagents and recombinant standards produced in mammalian cells.
• The multiplex assay quantified all 3 cytokines across a broad concentration range in plasma and cell culture supernatants.

## Graphical Abstract

**Figure:**
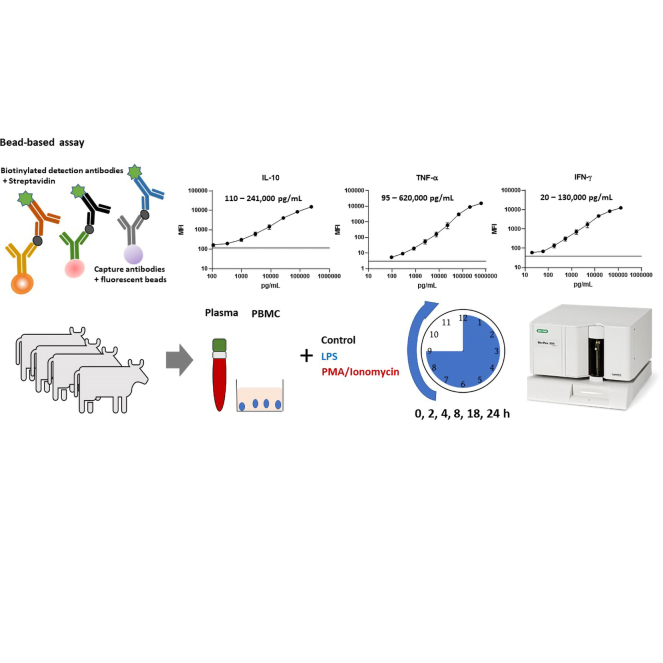
**Summary:** Bead-based assays provide a high-throughput platform allowing for simultaneous quantification of multiple cytokines. Our objective was to develop a multiplex bead-based assay using monoclonal reagents for simultaneous quantification of bovine tumor necrosis factor (TNF)-α, IL-10, and IFN-γ in plasma and peripheral blood mononuclear cell (PBMC) culture supernatants. Recombinant cytokine standards produced in mammalian cells were used to determine the lower limit of detection and linear detection range for each cytokine. Concentrations of native cytokines were determined in PBMC culture supernatants (n = 4) and plasma from whole-blood samples (n = 6) with or without stimulation with LPS or a mix of phorbol myristate acetate (PMA) and ionomycin. The multiplex assay can quantify native IL-10, TNF-α, and IFN-γ across a broad concentration range in bovine plasma and cell culture samples, thereby providing a novel tool to evaluate inflammatory profiles in cattle, especially in dairy cows with inflammatory conditions.

Specific detection and quantification of bovine cytokines are essential in understanding the effect of inflammatory mediators on dairy cow health and productivity, and in describing effects when mitigating inflammation (Bradford and Swartz, 2020). Proinflammatory cytokines such as tumor necrosis factor (**TNF**)-α and IFN-γ activate immune cell migration, antimicrobial function, and cytokine production in myeloid and lymphoid cells to initiate host defense, whereas anti-inflammatory or regulatory cytokines such as IL-10 restore homeostasis and inhibit proinflammatory cytokines (Coussens et al., 2012; Brodzki et al., 2015; Watari et al., 2019). Capturing the dynamics of proinflammatory and regulatory cytokines can provide valuable information to characterize the immune response in dairy cows and inform management decisions during inflammatory conditions.

Cytokine measurements by bead-based multiplex assays have several advantages over ELISA technology. The bead-based multiplex technology relies on microspheres (beads) with a single spectral address (color code; https://www.luminexcorp.com/). Each target-specific capture antibody is coupled to a color-coded bead, resulting in a unique fluorescence for each target. After a wash, biotinylated detection antibodies specific for each target are added, followed by another wash and incubation with the reporter dye streptavidin-phycoerythrin. Thus, each target is sandwiched between a unique, bead-coupled capture antibody and a biotinylated detection antibody plus reporter dye. The system applies a dual laser to identify the spectral signature of each bead and to simultaneously capture the intensity of the reporter associated with the bead. This allows for identification and quantification of multiple targets in a single run, thus saving sample volume and time. This technology has the potential for largely automated, high-throughput performance, facilitating timely analysis of large data sets (Wagner and Freer, 2009; Christopher-Hennings et al., 2013). Furthermore, compared with conventional ELISAs, the analytic sensitivity is improved 10- to 100-fold and the linear detection range of bead-based assays is about 1,000-fold wider (Wagner and Freer, 2009). Overall, bead-based multiplex assays allow for accurate quantification of cytokines in a variety of sample types and across wide concentration ranges, including detection of physiological concentrations of inflammatory markers, which can often be in the low picogram per milliliter range.

Only a few multiplex assays have been described for the detection of bovine cytokines. Dernfalk et al. (2007) developed bead-based single and multiplex assays for the detection of bovine IL-1β, IL-6, and TNF-α with a mix of mono- and polyclonal reagents in bovine plasma and milk. The limit of detection for this multiplex assay was in the nanogram per milliliter range. Rodrigues and colleagues (2017) developed a bead-based multiplex assay for quantification of bovine IFN-γ, IL-4, IL-10, IL-12, and TNF-α with monoclonal reagents in cell culture supernatants by flow cytometry. The lower detection limit for the targets ranged between 80 and 400 pg/mL. Both assays relied on protein standards of nonmammalian origin.

Our objective was to establish a bead-based multiplex assay by utilizing previously described mAbs for bovine TNF-α, IL-10, and IFN-γ and to quantify these 3 cytokines simultaneously in plasma and PBMC culture supernatants using recombinant bovine standards produced in mammalian cells.

We chose the following mAbs for coupling to different fluorescent beads (MicroPlex Microspheres, Luminex Corp.): anti-bovine TNF-α 197-1 (IgG_1_; Sipka et al., 2021) to bead 32; anti-bovine IL-10 CC318 (IgG_2b_, MCA2110, BioRad; Kwong et al., 2002) to bead 33; and anti-bovine IFN-γ CC330 (IgG_1_, MCA2112, BioRad; Baszler et al., 2008) to bead 35. All coupling and detection mAbs were developed in mice. Coupling of mAbs to fluorescent beads was performed according to the manufacturer's instructions and as previously described in Wagner and Freer (2009). Briefly, beads were initially activated with 100 m*M* sodium phosphate buffer, *N*-hydroxysulfosuccinimide (50 mg/mL), and 1-ethyl-3-[3 dimethylaminopropyl]carbodiimide hydrochloride (50 mg/mL, all Pierce Biotechnology). Subsequently, 100 μg of each mAb was coupled using 5 × 10^6^ activated beads over 3 h at room temperature and under rotation. The bead-coupled mAbs were then incubated with blocking buffer (**PBN**, PBS plus 1% BSA and 0.05% sodium azide) for 30 min at room temperature, washed 3 times with PBS plus 0.1% BSA, 0.05% sodium azide, and 0.02% Tween 20 (**PBS-T**), counted in a Neubauer hemocytometer, and stored at 4°C in the dark. The following biotinylated mAbs were used as detection antibodies in the assay: anti-bovine TNF-α 65-2 (IgG_1_; Sipka et al., 2021), anti-equine IL-10 492-2 (IgG_1_; Wagner et al., 2008), and anti-bovine IFN-γ CC302 (IgG_1_, MCA1783B, BioRad; Johnson et al., 2008). Specificity of the mAbs used in the assay was previously described by our group or others. The bead-coupled antibody for IL-10 was shown to neutralize the effect of recombinant bovine IL-10 and inhibited IFN-γ synthesis in antigen-stimulated bovine lymphocytes (Kwong et al., 2002). The anti-equine antibody used for detection of IL-10 was shown to be cross reactive with the bovine target in flow cytometric evaluation with bovine lymphocytes after stimulation with PMA and ionomycin (data not shown). The IFN-γ antibody pair was shown to detect the cytoplasmatic protein in flow cytometric analysis in bovine γδ T cells in response to IL-12 and IL-18 stimulation and in T-cell populations after experimental infection of cattle with *Neospora caninum* (Baszler et al., 2008; Johnson et al., 2008). The antibody pair detecting TNF-α was previously shown to selectively detect cytoplasmatic protein in CD14^+^ mononuclear cells after in vitro stimulation with LPS. Detection in lymphoid cell populations was only observed after PMA and ionomycin stimulation (Sipka et al., 2021). Each of the antibodies was described to bind to the native protein under experimental conditions involving bovine leukocyte populations expected to express the respective cytokine. In addition, all antibody pairs were tested in flow cytometric analysis for cytoplasmatic detection of their respective target. This was done using the Chinese hamster ovary (CHO) cell line that we used for production of the recombinant protein standard (see methods below). These findings confirmed that all mAbs could detect the native protein for each target.

For quantification of the cytokines in the multiplex assay, recombinant bovine cytokine/IgG fusion proteins were expressed by CHO cells and used as standards. Cells were transfected with mammalian expression vectors (pcDNA3.1, Invitrogen) containing the coding sequence of each bovine cytokine linked to the constant heavy chain region gene of equine IgG_1_ (IGHG1) as previously described (Wagner et al., 2005). The coding sequences (cDNA) for bovine TNF-α (GenBank: NM173966.3, bases 194–898), bovine IL-10 (GenBank: EU276074.1, bases 1–537), and bovine IFN-γ (GenBank: EU276066.1, bases 24–524) were amplified from bovine peripheral blood mononuclear cells (**PBMC**) and cloned into the expression vector as previously described (Sipka et al., 2021). To obtain the concentrations of the fusion proteins, we quantified the equine IgG_1_ portion of the fusion protein in an IgG_1_-specific assay and calculated the concentration of the recombinant bovine proteins based on their molecular weight by assuming an IgG_1_-to-cytokine ratio of 1:1 (Wagner et al., 2005).

The multiplex assay was set up as previously described (Wagner and Freer, 2009). Briefly, the bead-coupled capture mAbs were individually sonicated for 30 s, mixed, and diluted in PBN to a final concentration of 1 × 10^5^/mL, achieving a concentration of 5 × 10^3^ beads for each mAb and microtiter well in the assay. The cytokine standard mix contained all 3 recombinant bovine fusion proteins and was prepared in PBN in a series of 3-fold dilutions. Filter plates (MultiScreen HTS, Millipore) were soaked in PBS-T for 2 min. A plate washer (ELx50, BioTek) was used to aspirate the PBS-T and for all further wash steps. Then, we added 50 μL of the standard dilutions, sample, or PBN buffer (background value) to the plate followed by 50 μL of the bead mix. Subsequently, plates were incubated for 30 min at room temperature on a shaker. After incubation, plates were washed 3 times with the plate washer. These conditions were used for all subsequent incubation steps. Then, we added 50 μL of detection mAb mix diluted in PBN to each well followed by incubation and a wash. Finally, 50 µL of streptavidin R-PE (ThermoFisher Scientific) was added to all wells, and plates were incubated and washed as described above. For read-out, we added 100 μL of PBN to each well and the plate was placed in an automated reader (BioPlex 200, BioRad). Data were reported as mean fluorescent intensity (**MFI**), and the standard curve for all cytokine concentrations was fitted using the logistic *5p* formula (Wagner and Freer, 2009).

The quantification range was determined by serial dilutions of the standard fusion proteins and background values from assays run only with sample diluent (Figure 1A). Lower detection limits for the respective cytokines were 110 pg/mL for IL-10, 95 pg/mL for TNF-α, and 20 pg/mL for IFN-γ. The linear quantification range for IL-10 was 110 to 241,000 pg/mL, for TNF-α 95 to 620,000 pg/mL, and for IFN-γ 20 to 130,000 pg/mL (Figure 1A).

**Figure 1:**
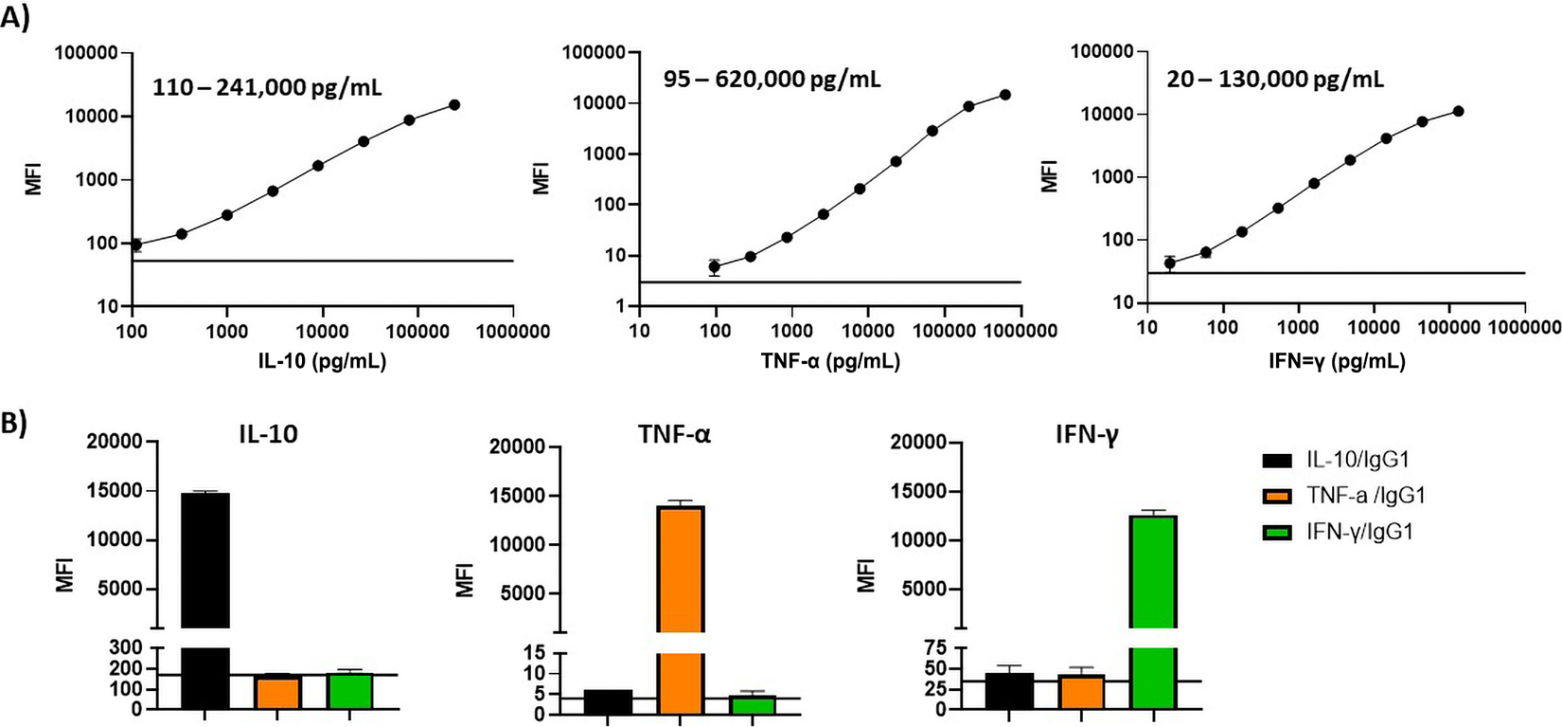
Linear range of detection, background values and cross reactivity test of the bead-based multiplex assay using fusion protein standards. (A) Serial dilutions of the IL-10, tumor necrosis factor (TNF)-α, and IFN-γ IgG_1_ fusion protein standards were prepared in assay buffer, and background values were obtained from running the multiplex assay with buffer only. Standard values are shown as mean fluorescence intensities (MFI) plus 95% CI from 12 independent runs. Background MFI is shown as the solid black line. (B) Cross reactivity between analytes of the multiplex assay was tested using the IL-10/IgG_1_, TNF-α/IgG_1_, and IFN-γ/IgG_1_ fusion protein standards. Each standard was analyzed separately in the otherwise complete multiplex assay. Values are shown as MFI plus 95% CI from 3 independent runs. Background MFI is shown as the solid black line.

We analyzed potential cross-reactivity between the 3 individual cytokine assays in the multiplex assay by testing complete mixtures of anti-IL-10, TNF-α, and IFN-γ coupled beads and detection mAbs with only one of the recombinant bovine cytokine fusion proteins, respectively (Figure 1B). Each cytokine was only detected by the corresponding bead assay, generating MFIs similar to those observed in the multiplex assay runs (Figure 1A). The MFIs of the other 2 cytokine assays were comparable with background values, demonstrating that there was no cross-reactivity between the 3 cytokine assays (Figure 1B).

Next, native secretion of IL-10, TNF-α, and IFN-γ was evaluated in bovine whole-blood cells and PBMC culture supernatants using the multiplex assay.

Sampling procedures in cows were approved by the Institutional Animal Care and Use Committee at Cornell University (Ithaca, NY; IACUC protocol 2017–0107). For whole-blood stimulation, blood samples from adult lactating cows were obtained (n = 6, 100–250 DIM) by venipuncture of the coccygeal vessels and collection of blood into evacuated glass tubes with sodium heparin as anticoagulant (BD Vacutainer, Becton Dickinson). For stimulation of PBMC, cells from heparinized whole blood of lactating cows (n = 4; 100–250 DIM) were separated by density gradient centrifugation as previously described (Sipka et al., 2016). We stimulated whole-blood samples and PBMC (5 × 10^6^ cells/mL) with *Escherichia coli* LPS (O111:B4, 100 ng/mL) or a mix of phorbol myristate acetate (**PMA**, 25 ng/mL) and ionomycin (750 ng/mL, all from Millipore Sigma), or left them as unstimulated controls. Whole blood was incubated at 38°C for 2, 4, 8, 18, and 24 h. Plasma from 2 mL of blood was harvested immediately as a preincubation control sample. Plasma was harvested by centrifugation for 10 min at 1,500 × *g* and 4°C. In parallel, PBMC were incubated at 37°C in a 5% CO_2_ enriched atmosphere for the same time points as whole-blood samples. Cell culture supernatants were collected by centrifugation for 10 min at 250 × *g* and 4°C. All samples were stored at −80°C until analyzed. Before analyzing in the bead-based assay, plasma samples were centrifuged at 13,000 × *g* for 5 min at room temperature to remove fibrin and debris.

All samples were analyzed in singlets. Intra- and interassay coefficients of variation (**CV**) for each analyte in the linear quantification range were calculated by dividing the standard deviation of the samples by the sample mean and expressed as percentages. Intraassay CVs for PBMC cell culture supernatants and plasma, respectively, calculated from 9 replicates, were 6.0% and 3.0% for IL-10, 4.9% and 9.0% for TNF-α, and 7.6% and 7.0% for IFN-γ. Interassay CVs for PBMC culture supernatants and plasma, respectively, were calculated from 1 replicate across 12 independent runs, and were 4.9% and 11.8% for IL-10, 8.4% and 9.4% for TNF-α, and 6.4% and 9.5% for IFN-γ. Additionally, the performance of the standard curve and the background values over time were monitored for all targets and runs in a Levey-Jennings control chart (data not shown). We applied a control limit of the MFI ± 2 standard deviations for each new run against historical data.

In PBMC culture supernatants, all cytokine concentrations increased over time in response to all treatments, including some spontaneous secretion in the unstimulated control sample (Figure 2A). Both PMA/ionomycin and LPS are known to induce production of bovine IL-10, TNF-α, and IFN-γ in PBMC (Jacobsen et al., 2007; Sassu et al., 2020). We observed the highest concentrations of all 3 cytokines in PBMC cultures in response to PMA/ionomycin stimulation. This was expected because PMA/ionomycin broadly stimulates cytokine production in all PBMC (Rossol et al., 2011). We measured peak secretion of TNF-α in response to PMA/ionomycin at 8 h, and TNF-α concentration plateaued afterward. Peak concentrations of IL-10 and IFN-γ were reached at 18 and 24 h, respectively. Spontaneous production of IL-10, TNF-α, and IFN-γ in unstimulated PBMC in vitro after 5 to 24 h of incubation has been observed by others (Friberg et al., 1994; Walker et al., 2002). Spontaneous cytokine secretion could have been triggered by damage-associated molecular patterns (DAMPs) or other stressors associated with cell death during culture (Maslanik et al., 2013).

**Figure 2:**
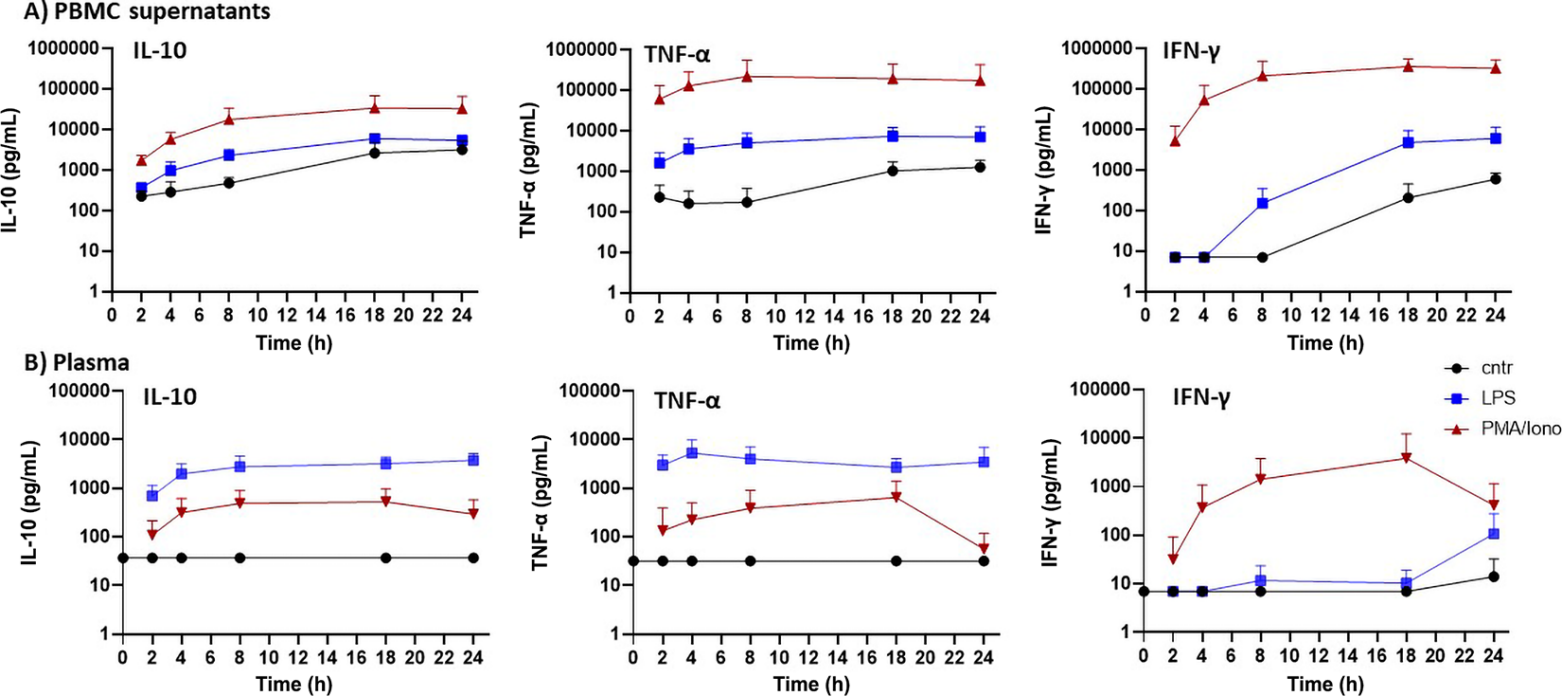
Quantification of bovine IL-10, tumor necrosis factor (TNF)-α, and IFN-γ in cell culture supernatants from peripheral blood mononuclear cells (PBMC) and plasma from whole-blood cell stimulations using a bead-based multiplex assay. Whole blood and PBMC were stimulated with LPS (100 ng/mL) or a mix of phorbol myristate acetate (PMA; 25 ng/mL) and ionomycin (Iono; 750 ng/mL), or left as unstimulated control (cntr). (A) PBMC (n = 4) or (B) whole-blood samples (n = 6) were stimulated for 0, 2, 4, 8, 18, or 24 h. Data are shown as mean plus 95% CI. Values below the detection limit are shown as the value of the lowest standard curve point divided by 3.

Concentrations of all 3 cytokines in plasma samples from whole-blood stimulation were below the detection limit in the 0-h sample and in the unstimulated control samples at all time points (Figure 2B). Reports on baseline concentrations of bovine IL-10, TNF-α, and IFN-γ plasma vary considerably across the literature, from below the detection limit (Dernfalk et al., 2007; Cousillas-Boam et al., 2020) to values in the low to mid picogram per milliliter range (Sacchini et al., 2012; Brodzki et al., 2015). It is impossible, however, to compare these findings because of the differences in reagents (polyclonal vs. monoclonal), recombinant proteins used as standards, and missing information on assay validation from some commercially available ELISA kits. We observed the highest concentration of TNF-α in plasma in response to LPS stimulation at 4 h, whereas IL-10 concentrations continuously increased until 24 h. Very low concentrations of IFN-γ were observed in response to LPS after 24 h of stimulation. Previous work has shown that production of TNF-α and IL-10 can be triggered in myeloid cells in response to LPS, while IFN-γ is produced mainly by lymphoid cells following other stimulants and can be suppressed by the presence of IL-10 (Rossol et al., 2011; Sheridan et al., 2017). Thus, stimulation of ex vivo whole blood with LPS is likely geared toward predominantly myeloid cells. After stimulation of whole-blood cell samples with PMA/ionomycin, comparable concentrations of all 3 cytokines were detected at all time points, which was expected due to the strong and general effect of the protein kinase C activator PMA and the calcium ionophor ionomycin on cell activation and cytokine production across all leukocytes in whole blood (Crawford et al., 2014; Schnabel et al., 2018).

The pattern of cytokine secretion in supernatants from bovine PBMC and plasma from whole-blood stimulation strongly suggests that the assays detected their respective native target cytokines. In addition, we prepared serial dilutions of supernatants from PMA/ionomycin–stimulated PBMC and plasma from LPS-stimulated whole blood and compared them with serial dilutions of the protein standards (Figure 3). The dilution series of the different samples showed acceptable parallelism, indicating that the bead-based multiplex assay can accurately quantify IL-10, TNF-α, and IFN-γ in PBMC culture supernatant or plasma within the broad linear detection ranges of the assay.

**Figure 3:**
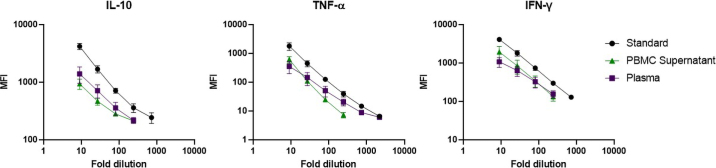
Serial dilution of samples and standards within the linear quantification range of the multiplex bovine cytokine assay. Serial dilutions of the IL-10/IgG_1_, tumor necrosis factor (TNF)-α/IgG_1_, and IFN-γ/IgG_1_ standards, 24-h stimulated peripheral blood mononuclear cells (PBMC) culture supernatants (n = 4; 25 ng/mL phorbol myristate acetate and 750 ng/mL ionomycin), and plasma from 24-h whole-blood stimulation (n = 4; 100 ng/mL *Escherichia coli* LPS) were analyzed in parallel. Data are shown as mean fluorescence intensity (MFI) plus 95% CI for 3-fold serial dilutions.

In conclusion, we developed a bead-based multiplex assay for bovine IL-10, TNF-α, and IFN-γ with lower detection limits in the low to mid picogram per milliliter range, high precision, and no cross-reactivity for the 3 cytokine targets. The new bead-based multiplex assay quantified bovine IL-10, TNF-α, and IFN-γ in a broad concentration range in cell culture supernatants and plasma from ex vivo whole-blood stimulation. The assay can be a useful tool to evaluate inflammatory profiles in dairy cows. Furthermore, the bead-based detection mechanism allows the existing assay to be expanded to detect more cytokines and chemokines and create a comprehensive platform for bovine inflammation markers.
